# miRFam: an effective automatic miRNA classification method based on n-grams and a multiclass SVM

**DOI:** 10.1186/1471-2105-12-216

**Published:** 2011-05-28

**Authors:** Jiandong Ding, Shuigeng Zhou, Jihong Guan

**Affiliations:** 1 School of Computer Science, Fudan University, Shanghai 200433, China; 2 Shanghai Key Lab of Intelligent Information Processing, Shanghai 200433, China; 3 Department of Computer Science & Technology, Tongji University, Shanghai 200433, China

## Abstract

**Background:**

MicroRNAs (miRNAs) are ~22 nt long integral elements responsible for post-transcriptional control of gene expressions. After the identification of thousands of miRNAs, the challenge is now to explore their specific biological functions. To this end, it will be greatly helpful to construct a reasonable organization of these miRNAs according to their homologous relationships. Given an established miRNA family system (e.g. the miRBase family organization), this paper addresses the problem of automatically and accurately classifying newly found miRNAs to their corresponding families by supervised learning techniques. Concretely, we propose an effective method, *miRFam*, which uses only primary information of pre-miRNAs or mature miRNAs and a multiclass SVM, to automatically classify miRNA genes.

**Results:**

An existing miRNA family system prepared by miRBase was downloaded online. We first employed *n*-grams to extract features from known precursor sequences, and then trained a multiclass SVM classifier to classify new miRNAs (i.e. their families are unknown). Comparing with miRBase's sequence alignment and manual modification, our study shows that the application of machine learning techniques to miRNA family classification is a general and more effective approach. When the testing dataset contains more than 300 families (each of which holds no less than 5 members), the classification accuracy is around 98%. Even with the entire miRBase15 (1056 families and more than 650 of them hold less than 5 samples), the accuracy surprisingly reaches 90%.

**Conclusions:**

Based on experimental results, we argue that *miRFam *is suitable for application as an automated method of family classification, and it is an important supplementary tool to the existing alignment-based small non-coding RNA (sncRNA) classification methods, since it only requires primary sequence information.

**Availability:**

The source code of *miRFam*, written in C++, is freely and publicly available at: http://admis.fudan.edu.cn/projects/miRFam.htm.

## Background

Sequences of DNA, RNA and proteins are the fundamental currency of modern biological research, which link the different levels of the biological hierarchy, from genes to 3D structures [1]. Common features of species and functionally important residues can be identified through sequence mining. RNA, which stores information like DNA and acts as an enzyme like proteins, may have supported cellular or pre-cellular life [2], and is crucial to protein synthesis that plays a very important role in life.

**Figure 1:**
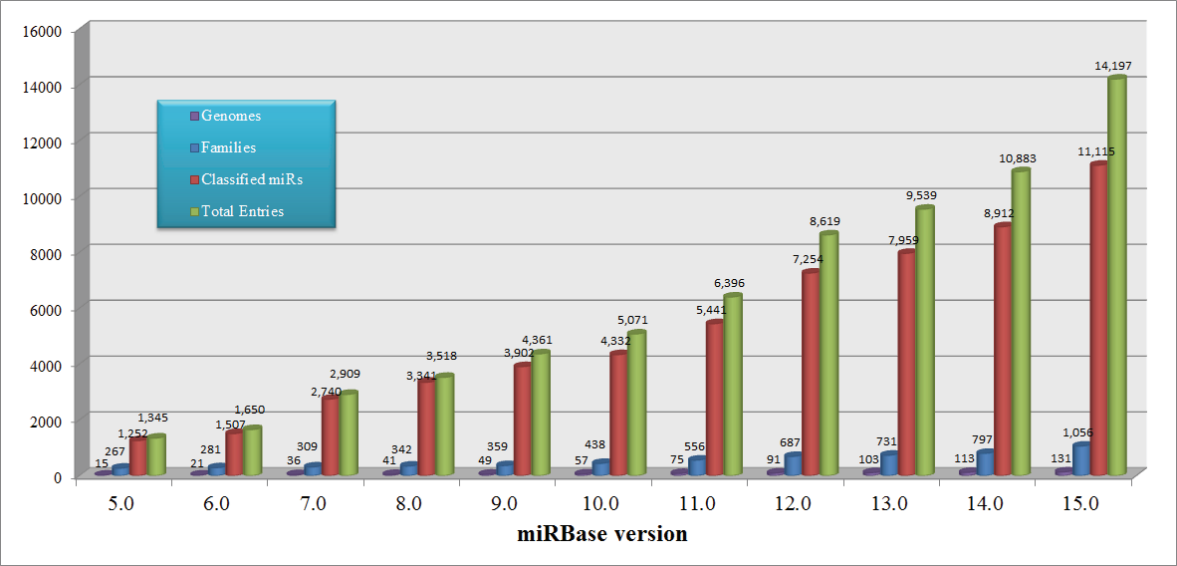
**The explosion of miRNA genes (Sep. 2004 - Apr. 2010)**. MiRNAs registered in miRBase increased rapidly in recent years. Almost at the same time when we finalized this manuscript, the 16th version of miRbase was released on 10 September 2010. Here, the latest information is not shown. Similar information was also exhibited in [10].

There are many RNAs with other roles in particular regulation of gene expression. Research shows that non-coding RNA genes produce a functional RNA product rather than a translated protein [3]. The most startling recent development in the non-coding RNA (ncRNA) field is the widespread importance of microRNA (miRNA). In the past six years, accompanied with the development of experimental [4,5] and computational [6-9] miRNAs detecting methods, the number of miRNA genes registered in miRBase [10] increased rapidly. We explored miRBase from version 5 to version 15 and found that the number of known miRNAs increased rapidly during the last several years (Figure 1). A similar trend can also be seen in [10]. It can be expected that with the use of next-generation sequencing technology [11-13], more miRNA genes will be identified. MiRNAs [14], belonging to the family of small non-coding RNAs (sncRNAs), are endogenous in many animal and plant genomes, and are now recognized as one of the major regulatory gene families in eukaryotic cells [15]. They modulate diverse biological processes, including embryonic development, tissue differentiation, and tumorigenesis. MiRNAs inhibit translation and promote mRNA degradation via sequence-specific binding to the 3'UTR regions of mRNAs [16]. Mature miRNAs are derived from longer precursors, each of which can fold into a hairpin structure that contains one or two mature miRNAs in either or both its arms [17]. The biogenesis of a miRNA in animals consists of two steps. In the first step, the primary miRNA (pri-miRNA), which is several hundred nucleotides long, is processed in the nucleus by a multi-protein complex containing an enzyme called *Drosha *to give rise to the ~70 nt long miRNA stem-loop precursor (pre-miRNA), which is then exported to the cytoplasm. The second step takes place in the cytoplasm where the pre-miRNA matures into a ~22 nt long miRNA:miRNA* duplex, with each strand originating from the opposite arm of the stem-loop [18]. Then, the miRNA strand of the miRNA:miRNA* duplex is loaded into a ribonucleoprotein complex known as the miRNA-induced silencing complex (miRISC) [19]. To date, the miRNA* was thought to be peeled away and degraded. However, some studies indicate that miRNA* is also sorted into Argonauts and might have a regular function in *Drosophila melanogaster *[20].

MiRBase is the central online repository of miRNA nomenclature, sequence data, annotation and target prediction, which first appeared in Oct. 2002 [21]. Release 15 contains 14197 miRNA loci from 66 species. From version 5.0, miRBase began to classify miRNAs into different families.

This kind of information was stored in *miFam.dat*, which was freely available online http://www.mirbase.org. These families were prepared manually. Essentially, it was done by using the single-linkage method to cluster the precursor sequences based on BLAST hits, and then adjusting (merging and/or splitting) manually the clustered families by multiple sequence alignment. The aim is to put miRNAs that have a common ancestor into the same family.

Rfam [22] is another well known RNA database. It contains a collection of multiple sequence alignments and *covariance models *(CMs) that represent ncRNA families. The primary aim of Rfam is to annotate new members of known RNA families on nucleotide sequences, particularly complete genomes, by using sensitive BLAST filters in combination with CMs. Both primary sequences and base-paired secondary structures are used to establish and annotate families. Release 10 contains 1446 families, including 453 miRNA families. But the quality of multiple sequence alignments and secondary structures is still a challenge for Rfam. Furthermore, Rfam requires a lot of computing resources to establish the family structure, which is time consuming, especially when the number of sequences is huge.

Since pre-miRNAs can form stable hairpins, this specific structural property has been used to cluster or classify them by some ncRNA clustering or classification methods [23,24]. Will *et al. *[23] presented a structure-based clustering approach, LocARNA (local alignment of RNA), which is capable of extracting putative RNA classes from genome-wide survey of structured RNAs. The performance of LocARNA relies on the prediction accuracy of RNA secondary structures. However, current RNA secondary structure energy models are not always able to predict native RNA structures, even for short molecules [25]. Furthermore, hairpin secondary structure might be less effective in miRNA classification since all miRNAs can fold back into this type of structure.

By far, multiple sequence and/or structure alignments are still widely used in ncRNA clustering and classification field. But neither of them has completely solved the ncRNA clustering or classification problem, especially for miRNAs. Not to mention effectiveness, only efficiency is still far from being satisfactory, since these methods could be very time-consuming when applied to large-scale validation of miRNA families.

As we know, miRNAs are highly conserved in not only their primary sequences but also their secondary structures. And miRNAs in the same family always have consensus secondary structures and similar functions [26]. Hence, it is biologically significant to subsume miRNAs with consensus second structures and similar functions to the same family. In this paper, based on the family system provided by miRBase, we explored supervised learning techniques to accurately and automatically classify pre-miRNA or mature sequences.

Concretely, we propose an effective alignment free model named *miRFam *to classify newly detected miRNAs. First, it extracts *n*-grams as features from primary sequences. Then, these *n*-gram features are integrated into one feature vector by *concentration*. Finally, it trains a multiclass SVM classifier SVM*^multiclass ^*based on the families prepared by miRBase to classify new pre-miRNA or mature sequences whose families are not yet known.

As a powerful tool, *miRFam *aims to classify new miRNAs into their corresponding families. It can not only support researchers who just obtained novel miRNAs computationally or experimentally to go on exploring the function of these miRNAs, but also enhance the utility of miRBase by providing higher automation and accuracy for miRNA classification. When measuring sequence similarity, unlike BLAST [27] or other BLAST-based methods, *miRFam *uses shorter sequence segments, thus it has a much smaller search space, which allows it to run faster. As the first miRNA-oriented sncRNA family classification method, *miRFam *has several advantages: (1) Only primary information of miRNAs is required, no other assumption (e.g., common secondary structures within a family or limitation of sequence length) is imposed. (2) Compared with multiple sequence alignment (MSA), *miRFam *is more efficient and accurate. To classify ~10,000 pre-miRNA sequences, MSA will cost several hours while *miRFam *consumes only several minutes. (3) *miRFam *is insensitive to sequencing error and the exact position of pre-miRNA in pri-miRNA. The change of several bases has very little effect on the feature vectors.

## Results

**Figure 2:**
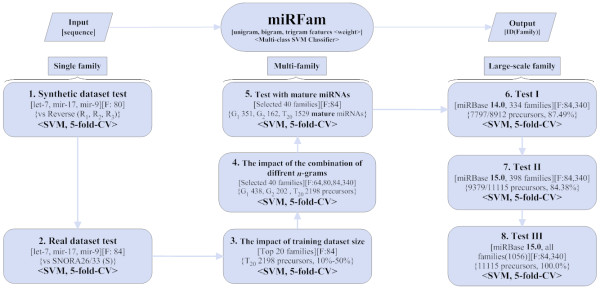
**The experimental pipeline**. In order to show the discriminative power of *miRFam*, we designed a series of experiments including single family tests, multi-family tests, and tests on large-scale miRBase families. All these experiments were carried out by 5-fold cross validation. Details of datasets and features were also shown.

In order to evaluate the *miRFam *method, we designed a pipeline that is illustrated in Figure 2. The experiments were arranged into three groups: single family tests, multi-family tests and application-oriented large-scale miRBase family tests, which were conducted on a number of datasets whose details are presented in the methods section. We started with single family tests, then multi-family tests and finally application-oriented large-scale miRBase family tests. Single family tests are classical binary classification, while the other tests are multi-class classification. With *miRFam*, users can conveniently choose different combinations of *n*-grams. According to our experience, unigrams, bigrams, trigrams and tetragrams are enough to classify all miRNAs registered in miRBase. For single family and multiple-family tests, even only unigrams, bigrams and trigrams are enough to achieve satisfactory classification performance. All experimental results were achieved by 5-fold cross validation. That is, each dataset is first randomly divided into five equally-sized partitions, each of which contains the same ratio of positive and negative examples. And then any four partitions are merged as the training set to train *miRFam*, which is further evaluated with the fifth data partition. This procedure is repeated five times with different combinations of training and testing sets, and the final classification performance is obtained by averaging the five tests' results.

### Single family tests

#### Synthetic dataset test

The three biggest families in miRBase14 are let-7, mir-17 and mir-9, which contain 208, 154 and 134 members, respectively. These three families were merged with three synthetic datasets R1, R2 and R3, respectively. *miRFam *was then tested on these three merged datasets, which are denoted as "let-7+R1", "mir-17+R2" and "mir-9+R3". Our aim is to show that *miRFam *can distinguish real pre-miRNAs from synthetic random sequences with similar base compositions. As expected, the combination of *n*-gram and multiclass SVM algorithm can precisely classify real miRNAs and random sequences. Experimental results are presented in Table 1, from which we can see that the accuracy is higher than 98.5% for all three families. Next, we took "let-7+R1", which gets the middle accuracy, as an example for further analysis. In 5-fold cross validation, only four sequences (MI0010673, MI0010668, RANDOM195, RANDOM198) were misclassified. MI0010673 and MI0010668 were first discovered from *Schistosoma japonicum *by cloning and sequencing a small (18-26 nt) RNA cDNA library from adult worms [28]. We submitted these two real miRNA sequences to Rfam (version 10.0) separately, but no hit was obtained. We then turned to *Clustal W2 *to generate the MSA with default parameters and viewed the guide tree by *Jalview2.5 *(see Figure S1 in additional file 1). We found that MI0010673 and MI0010668 were located in separate branches, while RANDOM195 and RANDOM198 lied in the nearby branches. Results showed that these synthetic sequences were so similar to the real ones that they were indistinguishable by using *miRFam *and MSA. In order to give a more intuitive picture of these datasets, we calculated the *Euclidean distance *(ED) between the real and synthetic cluster centers, and we found that the larger the *Euclidean distance *is, the better the classification performance is (see Figure S2 in additional file 1).

**Table 1:** Results of single family experiments

	experiment	SE(%)	SP(%)	Acc(%)
	let-7+R1	99.50	99.52	99.51
R*	mir-17+R2	100.0	100.0	100.0
	mir-9+R3	98.58	98.46	98.52

	let-7+S	99.02	99.69	99.42
S	mir-17+S	99.33	99.69	99.57
	mir-9+S	100.0	99.38	99.56

* Only trigram and bigram features are considered in these experiments.

#### Real dataset test

MiRNAs and snoRNAs are two classes of small non-coding regulatory RNAs, which have been extensively investigated in recent years. Although their functions in the cell are distinct, they share interesting genomic similarities. Recent sequencing projects have identified processed forms of snoRNAs that resemble miRNAs. A comparison of the genomic locations of reported miRNAs and snoRNAs reveals an overlap of some specific members of these two classes [29,30]. Keeping this in mind, we evaluated *miRFam *on another three datasets, which were constructed by merging dataset S with the families let-7, mir-17 and mir-9, and were denoted as "let-7+S", "mir-17+S" and "mir-9+S", respectively. The results are presented in Table 2, which shows that *miRFam *can easily distinguish miRNAs from snoRNAs, and the accuracies are higher than 99%.

**Table 2:** Results of different combinations of n-gram types

Group	Acc(trigram)	Acc(tri-&bigram)	Acc(tri-, bi-&unigram)*^a^*	Acc(tri-, bi-&unigram)*^b^*
T_20_	90.67	96.21	68.90	96.76
G_1_	93.61	98.40	87.63	98.86
G_2_	87.62	99.01	87.74	99.01
Total*^c^*	85.08	93.48	63.75	93.62

*^a ^*Results of *miRFam *with unigram, bigram and trigram, without concentration factor. *^b^*Results of *miRFam *with unigram, bigram and trigram, with concentration factor. *^c^*Combination of T_20_, G_1 _and G_2_. All results are percentiles.

#### The effect of concentration factor

In this paper, we introduced the concentration factor to weight the features of family vectors (see Equ. 2). Intuitively, the longer fragments of sequences should be more informative than the shorter ones. For example, with some exceptions [31], a triplet codon in a nucleic acid sequence specifies a single amino acid. And here, a trigram is exactly a triplet. Thus, in representing miRNAs sequences, the longer *n*-grams should outweigh the shorter ones. In what follows, we will see whether our concentration factor weighting scheme conforms to the above intuition and observation, by checking the centers (before and after weighting) of the three families (let-7, mir-17 and mir-9) and dataset S (the mixed snoRNA class).

**Figure 3:**
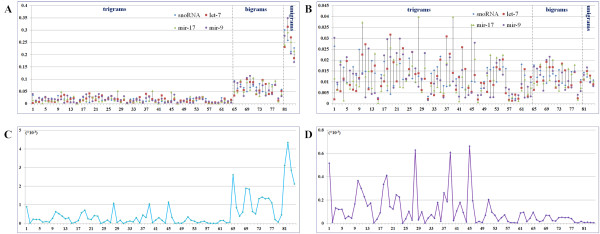
**Center vectors comparison among three miRNA families (let-7, mir-17 and mir-9) and the dataset S (snoRNAs)**. All horizontal axes are the *n*-grams arranged in the order from trigrams to bigrams and unigrams. (A) Family centers before weighting; (B) Family centers after weighting; (C) The variances of *n*-gram feature values among the four families before weighting; (D) The variances of *n*-gram feature values among the four families after weighting.

Figure 3(A) and Figure 3(B) are the center vectors before and after weighting (evaluated by  and  respectively) of four families. Roughly, before the weighting, trigrams have apparently smaller values than bigrams and unigrams. But after the weighting, trigrams get substantially enhanced. Furthermore, we calculated the variance of each feature's value among four families before and after weighting, the results are illustrated in Figure 3(C) and Figure 3(D). We can see that after weighting, the variances of trigrams are relatively enlarged, while the variances of bigrams and unigrams are substantially restrained. That is to say, our weighting scheme makes the trigram feature values of different families be more discrepant, which will benefit the classification of these families. Additionally, we evaluated the effect of concentration factor on multi-family datasets (Table 2). Without the concentration factor, more than 10% classification accuracy was lost on all datasets. *MiRFam *performed even worse when only trigrams were used.

In summary, the analysis on the feature vectors of different families shows that the concentration factor weighting scheme can enhance the trigrams while restraining the bigrams and unigrams, which is reasonable and consistent to the intuition and observation. Most importantly, our extensive classification experiments in this and the later sections also show indirectly that the weighting scheme is effective.

### Multi-family tests

As mentioned before, with the development of powerful deep sequencing technology, more miRNA genes will be identified in the future. But the number of real miRNAs in a certain genome is still unknown. Thus, a major concern is how well *miRFam *will perform if only a small number of known miRNAs are available for some certain families and species. In the previous single family tests, we have employed three types of *n*-grams (unigrams, bigrams and trigrams) as features, so one natural question is how the different combinations of these types of *n*-grams will impact *miRFam*'s performance. Furthermore, as mature miRNAs and hairpin sequences are somehow a little different, it occurs to us whether *miRFam *will perform differently on them. To answer these questions, we tested *miRFam *on three multi-family datasets constructed from miRBase (version 14) according to their family members. T_20 _contains the top 20 biggest families in miRBase (version 14), while G_1 _and G_2 _contain those families whose members are around 40 and 20, respectively. Here, the numbers 40 and 20 are randomly selected. Performance measurements like sensitivity and specificity are usually defined for binary classification. Here we actually deal with multi-class (i.e. multi-family) classification, so we use accuracy (Acc) as the performance indicator.

#### The impact of training dataset size

**Figure 4:**
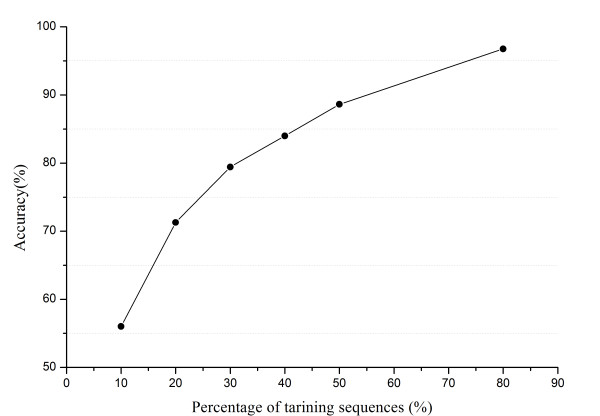
**Classification performance vs. the size of training dataset**. We used T_20 _to show the impact of training dataset size. At the beginning, only 10% of 2198 sequences in T_20 _were treated as training samples while others (90%) were used to test *miRFam*. At each round, we increased the training set by one partition (10%), and accordingly the testing set was reduced by one partition (10%). This process continued iteratively till half of T_20 _was for training and the other half for testing. The result of normal 5-fold crass validation is also shown.

All 2198 precursor sequences in T_20 _were divided into ten equally-sized partitions. First, we randomly took one partition (10%) of the sequences as the training set, the remaining nine partitions (90%) as the testing set. Then, we increased the training set by one partition (10%), and accordingly the testing set was reduced by one partition (10%). This process continues iteratively till half of T_20 _was for training and the other half for testing. At each round, *miRFam *was trained and tested, and its performance is evaluated by cross validation. As shown in Figure 4, the accuracy is 56.01% when only 10% of T_20 _is used for training. With the increase of training samples, the accuracy stably goes up. When the training set and the testing set are of equal size, the accuracy of *miRFam *is nearly 90%. For a normal 5-fold cross validation on the whole dataset, i.e, training *miRFam *with 80% samples and testing it with the remaining 20%, the accuracy is 96.76%.

#### The impact of the combination of different n-gram types

Here, we examine how classification performance will be impacted by the different combinations of unigrams, bigrams and trigrams on these multi-family datasets (Table 2). Actually, we also test *miRFam *with tetragrams, the results are presented in Additional file 1, Table S3.

We found that *miRFam *performs better when more types of *n*-gram features were used. Even when only the trigrams were used to classify miRNAs, the accuracy is around 90%. For the G_2 _dataset, when features of unigram, bigram and trigram types were all included, the accuracy was surprisedly more than 99%. Further exploring the classification results, we also found that some abnormal sequences with noise bases (not A, U, G and C) were also classified correctly in 5-fold cross validation (sequences are listed in Table S2 in Additional file 1), which means that *miRFam *is insensitive to base changes, such as single-nucleotide polymorphism (SNP) or sequencing error.

In addition, by transforming pre-miRNA sequences to feature vectors, both normal and abnormal sequences were handled in a similar process, thus avoiding the cumbersome addition, deletion and modification operations used in MSA.

#### Test with mature miRNAs

It has been shown that miRNAs are modified after maturation [32]. So, we also evaluated *miRFam *on mature miRNAs contained in these multi-family datasets (Table 3). Comparing to the results in Table 3, it can be seen that in most cases, *miRFam *performs better with mature miRNAs than with all miRNAs, which indicates that *miRFam *can accurately classify both hairpin and mature sequences. In fact, for a mature miRNA, the seed region is always much more functional than the other regions, it is the core functional region of its precursor. Thus, miRBase also prefers to put miRNAs with similar mature sequences into the same families. That is the reason why *miRFam *can achieve better performance with shorter maturity. It is also more efficient to classify mature miRNAs than to classify pre-miRNAs, since matures usually contain no more than 30% bases of their precursors.

**Table 3:** Results on mature miRNAs

Group	Families	Members*	Acc(tri-, bi-&unigram, %)
T_20_	20	1529	96.80
G_1_	10	351	97.71
G_2_	10	162	99.38
Total	40	2042	95.03

* Two reasons why the numbers of mature sequences in multi-family datasets are less than that in hairpins. First, different pre-miRNAs may generate similar mature miRNAs. Second, some pre-miRNAs contain several mature miRNAs, but only one is considered.

### Application-oriented large-scale families tests

A good model should not be data specific, instead it should be generally applicable. Although *miRFam *can achieve excellent results in single family tests and multi-family tests, what we really care about is its practical application performance. Based on this consideration, we evaluated *miRFam *on large-scale families from miRBase (version 14 and 15). Results are presented in Table 4.

**Table 4:** Performance of large-scale miRBase families test

	miRBase14	miRBase15	
Family number	334*^a^*	398*^a^*	1056*^b^*
MiRNA number	7797	9379	11115
Accuracy (%)*^c^*	89.21	88.91	85.09
Accuracy (%)*^d^*	98.18	97.97	90.66

*^a ^*Families in miRBase whose members are no less than 5.

*^b ^*All families in miRBase 15 are used.

*^c ^miRFam *results with uni-, bi- and trigram features.

*^d ^miRFam *results with uni-, bi-, tri- and tetragram features.

Since 5-fold cross validation was employed, families that contain less than 5 members were not considered at first. A detailed family distribution in miRBase could be found in Figure S3 in the additional file 1. From miRBase v14, the 334 families that contain no less than 5 members were selected, which hold 87.49% (7797/8912) pre-miRNA sequences of the whole database. On this dataset, *miRFam *achieved an accuracy of 98.18%.

When we were preparing this manuscript, miRBase (version 15) was released in April 2010. This is a significant update, with over 3000 new hairpin sequences and more than 4000 new mature sequences. From miRBase v15, 398 families were selected, each of which contains no less than 5 members. These families constitute 84.38% (9379/11115) hairpin sequences in the whole database. Even with such large-scale families, *miRFam *still got an accuracy of 97.97%.

When dealing with miRBase v15, there are still 1736 pre-miRNAs distributed in 658 families that were not processed (see Figure S3). Among them, 351 families have only 2 members. In the final experiment, we tested *miRFam *on the whole 1056 families in miRBase v15. For those families with less than 5 members, we randomly chose one member as the testing sample, and the remaining as training samples, *miRFam *still obtained an accuracy of 90.66%, which was a surprisingly satisfactory result, considering that classifying a dataset with a large number of classes and the extremely uneven distributions of members in these classes is a well-recognized challenging task.

## Discussion

Effectively classifying newly detected miRNAs to their corresponding families is helpful for their further functional analysis. However, only a few works have been done to address this issue, which is far from being established. Unlike existing alignment-based sncRNA clustering or classification methods [23,33,34], which can also be used to cluster or classify miRNAs, the proposed *miRFam *bases on supervised learning techniques, which is more general and effective. It does not require sequence- or structure-based alignment, thus it is free from the difficulty of choosing multiple parameters used in the alignment-based methods, and is also free from the quality issue of miRNA secondary structure prediction. Certainly, *miRFam *is not completely parameter-free, it still has to set two parameters, i.e., the feature vector length *l *and the trade-off between training error and margin *c*. Another advantage of the *miRFam *method is its efficiency, especially when the number of sequences is huge. Furthermore, *miRFam *can achieve satisfactory classification performance over the family system prepared by miRBase. Of all predictions made by *miRFam*, the accuracy is beyond 90%. Therefore, it can be used to replace the manual modification, which will greatly save time.

Most known miRNA sequences are evolutionary conserved [35], miRNA families may have consensus secondary structures [26], and the microRNA-target relationships are also conservative [36]. As people's interest in the miRNA world continuously grows, more and more datasets are going to appear. Correspondingly, there is an urgent need to classify the newly discovered miRNAs into their corresponding families according to sequence and/or structure similarities. With correct family classification, it is easier to elucidate the structures and functions of the new sequences, by using multiple sequence alignments. Apparently, more in-depth information can also be available, such as SNPs within pre-miRNAs and mature miRNAs [37].

One potential limitation of the proposed approach is that it relies on a prepared family classification structure. Actually, this is a common problem with classification - a supervised machine learning approach, and the quality of training sets significantly influences classification accuracy. To overcome this limitation, we can turn to clustering analysis, which is an unsupervised learning approach that can automatically group the miRNA sequences into different categories based on their characteristics of sequences and/or structures. We keep this issue as our future work.

## Conclusions

Sequence alignments are useful for the analysis of genomic data. For example, miRNA genes in newly sequenced organism can be detected based on their homology to genes in related and well-studied species [4,38]. Once homologous genes are detected, one can perform a MSA with the hope of establishing miRNA families. However, MSA is time consuming in doing this work, different MSA algorithms may build quite different alignments, and choosing an appropriate alignment algorithm is crucial to the performance of family classification.

In this article, we developed a new approach *miRFam *to accurately and automatically classify miRNA precursors by using *n*-grams and a multiple-class SVM classifier. To evaluate the *miRFam *method, we designed a pipeline, including single family tests, multi-family tests and large-scale families tests. Based on the experimental results, the following conclusions could be drawn:

1. *miRFam *can effectively distinguish synthetic random sequences and similar snoRNA sequences from real pre-miRNA sequences (Table 1).

2. Even with a small number of training samples, *miRFam *can still achieve a high accuracy. And with more types of *n*-gram features, *miRFam *can perform better (Table 2 & Figure 4).

3. Both precursors and mature miRNAs can be used to infer miRNA families. With shorter mature sequences, *miRFam *can achieve better classification result (Table 3).

4. When the dataset contains more than 300 families and each family holds no less than 5 members, the classification accuracy is around 98%. Even with the entire miRBase (version 15, 1056 families and more than 650 of them hold less than 5 samples), the accuracy surprisedly reaches 90% (Table 4).

In summary, we proposed the first supervised learning based approach *miRFam *to automatically assign miRNA precursors to their corresponding families with high accuracy. It can be useful to help family classification, especially in the applications that previously have been done manually, such as miRBase. Additionally, due to its robustness, *miRFam *can be used in a wide range of scenarios, as long as an existing family assignment information is available. Certainly, its performance depends on the existing family assignment information. However, as there is more and more study on miRNA, it is foreseeable that more miRNAs will be identified and registered in miRBase. Such a situation will certainly favor the existence and utilization of the *miRFam *method. In return, *miRFam *will also contribute a lot to the efficient exploration of these newly discovered miRNAs.

## Methods

### Datasets

In this work, we constructed several datasets using data from miRBase and Rfam. These datasets were divided into three categories: single family datasets, multi-family datasets and large-scale family datasets. To facilitate the description, we used some notations to represent the datasets of the first two categories. These notations are summarized in Table 5.

**Table 5:** Notations of datasets

	notation	description
Single family	R*^a^*	reverse sequences of the biggest three miRNA families
	S	combination of SNORA26 and SNORA33 from Rfam10.0

	T_20_	20 families with the largest members, ANM*^b ^*= 109.9
Multi families	G_1_	10 families selected from miRBase14, ANM*^b ^*= 43.8
	G_2_	10 families selected from miRBase14, ANM*^b ^*= 20.2

*^a ^*R1 - let-7; R2 - mir-17; R3 - mir-9.

*^b ^*ANM -- Average Number of Members.

We first ranked miRNA families in miRBase according to the number of members contained in each family. R contains three subsets R1, R2 and R3, corresponding to the three biggest families in miRBase v14 (let-7, mir-17 and mir-9). R1, R2 and R3 were constructed by reversing the original pre-miRNA sequences in let-7, mir-17 and mir-9 with *squid *[39], respectively. S was constructed by mixing SNORA26 and SNORA33 downloaded from Rfam v10.0.

SNORA26 (RF00568) is a member of the H/ACA class of small nucleolar RNAs, while SNORA33 (RF00133) is a member of the C/D box class. After being filtered to less than 90% identity, they contain 195 and 122 sequences, respectively. Three multi families datasets (T_20_, G_1_, G_2_) were constructed from miRBase v14 based on the result of family ranking. The biggest family in G_1 _is mir-33 containing 47 members, and the smallest family is mir-26 containing 41 members. While the biggest (smallest) families in G_2 _is mir-315 (mir-320), containing 21 (20) miRNAs (Additional file 1, Table S1).

### Feature vectors

In this paper, we treat family establishment as a classification problem. The first step is to transform miRNA sequences to numeric vectors, which are usually called feature vectors. Here, *n*-grams [40] are used as features of miRNA sequences.

#### n-grams

An *n*-gram is a subsequence consisting of *n *spatially consecutive items from a given sequence. The items in this study are pre-miRNA bases (A,C,G and U). A *n*-gram of size 1 (i.e. *n *= 1) is referred to as a "unigram", size 2 (*n *= 2) is a "bigram", size 3 (*n *= 3) is a "trigram", size 4 is a "tetragram", and size 5 or more (i.e. *n *≥ 5) is simply called a "*n*-gram". In the sequel, we also call unigrams, bigrams, trigrams and tetragram as type 1, 2, 3 and 4 *n*-grams, and so on. *n*-grams can be used for efficient approximate matching. By converting a miRNA precursor to a set of *n*-grams, it can be embedded into a vector space, thus allowing a sequence to be compared with others in an efficient manner. Here, we select unigrams, bigrams, trigrams and tetragram as features.

To extract *n*-grams, we use a window of size *n *that slides on pre-miRNA sequences from 5' to 3'. At each position on a sequence, the subsequence of length *n *covered by the sliding window corresponds to a *n*-gram. As the window slides forward, the occurrence frequency *t *of each encountered *n*-gram is recorded.

#### Concentration

Since RNA sequences contain only the four bases A, U, G and C, we have 4 unique unigrams, 4^2 ^unique bigrams, 4^3 ^unique trigrams and 4^4 ^unique tetragrams. In order to combine these different features into one feature vector, we introduce a *concentration *factor. Denote the number of unique *n*-grams of type *i *as *N_i_*. The concentration of type *i *is the ratio of *N_i_*over the total number of unique *n*-grams. That is,(1)

For example, the trigram (type 3) has 4^3 ^unique *n*-grams. The total number of unique *n*-grams used in this study is 340 (4+16+64+256), therefore trigram's concentration is C*_tri_*= 64/340 = 0.188. Then, the elements of a feature vector is calculated by (2).(2)

Above, *t_j _*is the occurrence frequency of a certain unique *n*-gram of type *i*, and *T_i _*is the total occurrence frequency of all unique *n*-grams of type *i*. A feature vector contains 340 dimensions, each of which corresponds to a unique *n*-gram of a certain type *i *(*i *= 1, 2, 3 and 4). Within a vector, the dimensions are arranged in the order of tetragrams, trigrams, bigrams and unigrams. The sum of all dimensional values of a feature vector is 1.

### Multiclass SVM

Binary classification using support vector machine (SVM) is a well developed technique. However, due to performance reasons, using a single SVM formulation directly to solve the multiclass problem is usually avoided. A better approach is to use a combination of several binary SVM classifiers to solve the multiclass problem. Typical algorithms of multiclass learning include the multiclass extensions to decision tree learning [41] and various specialized versions of the boosting approach such as AdaBoost.M2 and AdaBoost.MH [42,43]. However, the dominate approach to the multiclass problem is multiclass SVM. One of the most widely-used multiclass SVM methods is one-versus-all. In this method, *M *binary classifiers are constructed. The *i*-th classifier's output function *F_i_*is trained by using the examples from class *i *as positives and the examples from all other classes as negatives. For a new example *x*, the one-versus-all SVM strategy assigns it to the class with the largest value of *F_i_*[44].

In this study, we use the popular multiclass SVM package *SVM^multiclass ^*(version 2.20). *SVM^multiclass ^*uses the multi-class formulation described in [45], and is optimized so that it is very fast in linear cases [46].

### MSA implementation and visualization

Multiple sequence alignment is done by *Clustal W *(version 2.0) [47]. The tree visualization of MSA results is achieved by *Jalview *(version 2.5) [1]. These tools are also used by EMBL-EBI online.

### Evaluation

The most straightforward way to evaluate the performance of a classifier is based on the confusion matrix analysis. With this matrix, it is possible to evaluate a number of widely used metrics for measuring the performance of a learning system. Here, we use *sensitivity *(SE), *specificity *(SP) and *accuracy *(Acc) to evaluate *miRFam*. They are defined as follows:(3)

Here, *TP, FP, TN *and *FN *are the numbers of true positive predictions, false positive predictions, true negative predictions and false negative predictions, respectively.

## Conflict of interests

The authors declare that they have no competing interests.

## Authors' contributions

JD constructed the model, performed the experiments and prepared the manuscript. SZ and JG guided the research and scheme design, and helped to prepare and improve the manuscript. All authors read and approved the manuscript.

## Funding

This research was supported by Major State Basic Research and Development Program of China (973 Program) under grant no. 2010CB126604. JG was also supported by the Open Research Program of Shanghai Key Lab of Intelligent Information Processing.

## Supplementary Material

Additional file 1 **Supplement. We collect all supplementary tables and figures in this file**. The detailed family information and abnormal sequences contained in three multi-family datasets (T_20_, G_1_, and G_2_) can be found in Additional file 1, Table S1 and S2, respectively. Results of multi-family test with tetragram features are summarized in Additional file 1, Table S3. Figure S1 and S2 in Additional file 1 are supplied to support our analysis in Section "Synthetic dataset analysis", while Figure S3 in Additional file 1 shows the family distribution in miRBase (version 14 and 15) according to family member. Click here for file

## References

[B1] Waterhouse AM Procter JB Martin DMA Clamp M Barton GJ Jalview Version 2-a multiple sequence alignment editor and analysis workbench Bioinformatics 2009 25 9 1189 91 10.1093/bioinformatics/btp033 19151095 PMC2672624

[B2] Gilbert W Origin of life: The RNA world Nature 1986 319 6055 618 618

[B3] Griffiths-Jones S Annotating noncoding RNA genes Annual review of genomics and human genetics 2007 8 279 98 10.1146/annurev.genom.8.080706.092419 17506659

[B4] Lim LP Lau NC Weinstein EG Abdelhakim A Yekta S Rhoades MW Burge CB Bartel DP The microRNAs of Caenorhabditis elegans Genes & development 2003 17 8 991 1008 10.1101/gad.1074403 12672692 PMC196042

[B5] Grad Y Aach J Hayes GD Reinhart BJ Church GM Ruvkun G Kim J Computational and experimental identification of C. elegans microRNAs Molecular cell 2003 11 5 1253 63 10.1016/S1097-2765(03)00153-9 12769849

[B6] Rodriguez A Griffiths-Jones S Ashurst JL Bradley A Identification of mammalian microRNA host genes and transcription units Genome research 2004 14 10A 1902 10 10.1101/gr.2722704 15364901 PMC524413

[B7] Ng KLS Mishra SK De novo SVM classification of precursor microRNAs from genomic pseudo hairpins using global and intrinsic folding measures Bioinformatics 2007 23 11 1321 30 10.1093/bioinformatics/btm026 17267435

[B8] van der Burgt A Fiers MWJE Nap JP van Ham RCHJ In silico miRNA prediction in metazoan genomes: balancing between sensitivity and specificity BMC genomics 2009 10 204 10.1186/1471-2164-10-204 19405940 PMC2688010

[B9] Mathelier A Carbone A MIReNA: finding microRNAs with high accuracy and no learning at genome scale and from deep sequencing data Bioinformatics 2010 26 18 2226 2234 10.1093/bioinformatics/btq329 20591903

[B10] Kozomara a Griffiths-Jones S miRBase: integrating microRNA annotation and deep-sequencing data Nucleic Acids Research 2010 4 1 6 10.1093/nar/gkq1027 PMC3013655 21037258

[B11] Friedländer MR Chen W Adamidi C Maaskola J Einspanier R Knespel S Rajewsky N Discovering microRNAs from deep sequencing data using miRDeep Nature biotechnology 2008 26 4 407 15 10.1038/nbt1394 18392026

[B12] Hackenberg M Sturm M Langenberger D Falcón-Peréz JM Aransay AM miRanalyzer: a microRNA detection and analysis tool for next-generation sequencing experiments Nucleic acids research 2009 37 Web Server W68 76 10.1093/nar/gkp347 PMC2703919 19433510

[B13] Hendrix D Levine M Shi W miRTRAP, a computational method for the systematic identification of miRNAs from high throughput sequencing data Genome biology 2010 11 4 R39 10.1186/gb-2010-11-4-r39 20370911 PMC2884542

[B14] Lee RC Feinbaum RL Ambros V The C. elegans heterochronic gene lin-4 encodes small RNAs with antisense complementarity to lin-14 Cell 1993 75 5 843 54 10.1016/0092-8674(93)90529-Y 8252621

[B15] Jones-Rhoades MW Bartel DP Bartel B MicroRNAS and their regulatory roles in plants Annual review of plant biology 2006 57 19 53 10.1146/annurev.arplant.57.032905.105218 16669754

[B16] Liu J Control of protein synthesis and mRNA degradation by microRNAs Current opinion in cell biology 2008 20 2 214 21 10.1016/j.ceb.2008.01.006 18329869

[B17] Bartel DP MicroRNAs: genomics, biogenesis, mechanism, and function Cell 2004 116 2 281 97 10.1016/S0092-8674(04)00045-5 14744438

[B18] Zhang H Kolb FA Jaskiewicz L Westhof E Filipowicz W Single processing center models for human Dicer and bacterial RNase III Cell 2004 118 57 68 10.1016/j.cell.2004.06.017 15242644

[B19] Inui M Martello G Piccolo S MicroRNA control of signal transduction Nature reviews Molecular cell biology 2010 11 4 252 63 20216554 10.1038/nrm2868

[B20] Ghildiyal M Xu J Seitz H Weng Z Zamore PD Sorting of Drosophila small silencing RNAs partitions microRNA* strands into the RNA interference pathway RNA 2010 16 43 56 10.1261/rna.1972910 19917635 PMC2802036

[B21] Griffiths-Jones S The microRNA Registry Nucleic acids research 2004 32 Database D109 11 10.1093/nar/gkh023 PMC308757 14681370

[B22] Gardner PP Daub J Tate JG Nawrocki EP Kolbe DL Lindgreen S Wilkinson AC Finn RD Griffiths-Jones S Eddy SR Bateman A Rfam: updates to the RNA families database Nucleic acids research 2009 37 Database D136 40 10.1093/nar/gkn766 PMC2686503 18953034

[B23] Will S Reiche K Hofacker IL Stadler PF Backofen R Inferring noncoding RNA families and classes by means of genome-scale structure-based clustering PLoS computational biology 2007 3 4 e65 10.1371/journal.pcbi.0030065 17432929 PMC1851984

[B24] Griffiths-Jones S Bateman A Marshall M Khanna A Eddy SR Rfam: an RNA family database Nucleic acids research 2003 31 439 41 10.1093/nar/gkg006 12520045 PMC165453

[B25] Dowell RD Eddy SR Evaluation of several lightweight stochastic context-free grammars for RNA secondary structure prediction BMC bioinformatics 2004 5 71 10.1186/1471-2105-5-71 15180907 PMC442121

[B26] Kaczkowski B Torarinsson E Reiche K Havgaard JH Stadler PF Gorodkin J Structural profiles of human miRNA families from pairwise clustering Bioinformatics 2009 25 3 291 4 10.1093/bioinformatics/btn628 19059941

[B27] Altschul SF Gish W Miller W Myers EW Lipman DJ Basic local alignment search tool Journal of molecular biology 1990 215 3 403 10 2231712 10.1016/S0022-2836(05)80360-2

[B28] Xue X Sun J Zhang Q Wang Z Huang Y Pan W Identification and characterization of novel microRNAs from Schistosoma japonicum PloS one 2008 3 12 e4034 10.1371/journal.pone.0004034 19107204 PMC2603315

[B29] Ender C Krek A Friedländer MR Beitzinger M Weinmann L Chen W Pfeffer S Rajewsky N Meister G A human snoRNA with microRNA-like functions Molecular cell 2008 32 4 519 28 10.1016/j.molcel.2008.10.017 19026782

[B30] Scott MS Avolio F Ono M Lamond AI Barton GJ Human miRNA precursors with box H/ACA snoRNA features PLoS computational biology 2009 5 9 e1000507 10.1371/journal.pcbi.1000507 19763159 PMC2730528

[B31] Turanov AA Lobanov AV Fomenko DE Morrison HG Sogin ML Klobutcher LA Hatfield DL Gladyshev VN Genetic code supports targeted insertion of two amino acids by one codon Science 2009 323 5911 259 61 10.1126/science.1164748 19131629 PMC3088105

[B32] Morin RD O'Connor MD Griffith M Kuchenbauer F Delaney A Prabhu AL Zhao Y McDonald H Zeng T Hirst M Eaves CJ Marra MA Application of massively parallel sequencing to microRNA profiling and discovery in human embryonic stem cells Genome research 2008 18 4 610 21 10.1101/gr.7179508 18285502 PMC2279248

[B33] Griffiths-Jones S Saini HK van Dongen S Enright AJ miRBase: tools for microRNA genomics Nucleic acids research 2008 36 Database D154 8 10.1093/nar/gkm952 PMC2238936 17991681

[B34] Nawrocki EP Kolbe DL Eddy SR Infernal 1.0: inference of RNA alignments Bioinformatics 2009 25 10 1335 7 10.1093/bioinformatics/btp157 19307242 PMC2732312

[B35] Lee CT Risom T Strauss WM Evolutionary conservation of microRNA regulatory circuits: an examination of microRNA gene complexity and conserved microRNA-target interactions through metazoan phylogeny DNA and cell biology 2007 26 4 209 18 10.1089/dna.2006.0545 17465887

[B36] Chen K Rajewsky N Deep conservation of microRNA-target relationships and 3'UTR motifs in vertebrates, flies, and nematodes Cold Spring Harbor symposia on quantitative biology 2006 71 149 56 10.1101/sqb.2006.71.039 17381291

[B37] Meng Y Gou L Chen D Mao C Jin Y Wu P Chen M PmiRKB: a plant microRNA knowledge base Nucleic Acids Research 2010 38 16 1 7 20719744 10.1093/nar/gkq721 PMC3013752

[B38] Bentwich I Avniel A Karov Y Aharonov R Gilad S Barad O Barzilai A Einat P Einav U Meiri E Sharon E Spector Y Bentwich Z Identification of hundreds of conserved and nonconserved human microRNAs Nature genetics 2005 37 7 766 70 10.1038/ng1590 15965474

[B39] Rivas E Eddy SR Secondary structure alone is generally not statistically significant for the detection of noncoding RNAs Bioinformatics 2000 16 7 583 605 10.1093/bioinformatics/16.7.583 11038329

[B40] Manning C Schütze H Foundations of statistical natural language processing 2000 78 MIT Press

[B41] Quinlan JR C4.5: Programs for Machine Learning 1993 San Francisco, CA, USA Morgan Kaufmann Publishers Inc

[B42] Freund Y A Decision-Theoretic Generalization of On-Line Learning and an Application to Boosting Journal of Computer and System Sciences 1997 55 119 139 10.1006/jcss.1997.1504

[B43] Schapire RE Singer Y Improved boosting algorithms using confidence-rated predictions 1998 37 New York, New York, USA: ACM Press

[B44] Duan K Keerthi S Which is the best multiclass SVM method? An empirical study Multiple Classifier Systems 2005 3541 278 285 10.1007/11494683_28

[B45] Crammer K Singer Y On the Algorithmic Implementation of Multiclass Kernel-based Vector Machines Journal of Machine Learning Research 2002 2 2 265 292 10.1162/15324430260185628

[B46] Tsochantaridis I Hofmann T Joachims T Altun Y Support vector machine learning for interdependent and structured output spaces 2004 New York, New York, USA: ACM Press

[B47] Larkin MA Blackshields G Brown NP Chenna R McGettigan PA McWilliam H Valentin F Wallace IM Wilm A Lopez R Thompson JD Gibson TJ Higgins DG Clustal W and Clustal X version 2.0 Bioinformatics 2007 23 21 2947 8 10.1093/bioinformatics/btm404 17846036

