# Supplementary material for: miRFam: an effective automatic miRNA classification method based on n-grams and a multiclass SVM
Source: BMC Bioinformatics. 2011 May 28;12:216. doi: 10.1186/1471-2105-12-216 (PMC3120706; doi:10.1186/1471-2105-12-216)
Supplement: Additional file 1 — Supplement. We collect all supplementary tables and figures in this file. The detailed family information and abnormal sequences contained in three multi-family datasets (T20, G1, and G2) can be found in Additional file 1, Table S1 and S2, respectively. Results of multi-family test with tetragram features are summarized in Additional file 1, Table S3. Figure S1 and S2 in Additional file 1 are supplied to support our analysis in Section "Synthetic dataset analysis", while Figure S3 in Additional file 1 shows the family distribution in miRBase (version 14 and 15) according to family member. [file 1471-2105-12-216-S1.PDF]

Supplementary

miRFam: an effective automatic miRNA classification method based on n-grams and a multiclass SVM

Jiandong Ding[1, 2], Shuigeng Zhou[*1, 2] and Jihong Guan[*3]

[1]School of Computer Science, Fudan University, Shanghai 200433, China

[2]Shanghai Key Lab of Intelligent Information Processing, Shanghai 200433, China

[3]Department of Computer Science & Technology, Tongji University, Shanghai 200433, China.

* to whom correspondence should be addressed

Email:

Jiandong Ding - jdding@fudan.edu.cn;

Shuigeng Zhou - sgzhou@fudan.edu.cn;

Jihong Guan - jhguan@tongji.edu.cn;

Table S1: Family information of multi-family datasets (T_{20}, G_1 and G_2). Data comes from miRBase14 [1].

Group	Name	Member
T_{20}	*let-7*	208
	mir-17	154
	mir-9	134
	mir-154	128
	mir-25	123
	mir-2	122
	MIR166	114
	mir-515	113
	MIR156	106
	mir-15	102
	mir-29	101
	mir-8	97
	mir-506	93
	mir-30	92
	mir-181	91
	MIR395	89
	MIR171_1	87
	mir-124	82
	MIR159	81
	mir-7	81
	total	***2198***
G_1	*mir-33*	47
	mir-218	46
	mir-23	46
	MIR172	45
	mir-27	44
	mir-467	43
	mir-199	42
	MIR396	42
	mir-184	42
	mir-26	41
	total	***438***
G_2	*mir-315*	21
	mir-1302	21
	mir-96	20
	mir-105	20
	mir-217	20
	MIR445	20
	MIR398	20
	mir-375	20
	mir-450	20
	mir-320	20
	total	***202***

Table S2: Abnormal pre-miRNAs in multi-family datasets (T_{20}, G_1 and G_2). Data comes from miRBase14.

**T_{20}:**
>MI0012252   mir-9
UCGUCAUGGCGCUGCUUUUUUCUUUGGUUAUCUAGCUGUAUGAGUGUUAGAAGACGUCAUANA<br>GCUAGGUUACCAAAGUUAAGAACUGCCCCUCUUCAUCG
>MI0001487   MIR166
GGGGAAUGUUGUCUGGUUGGAGACCUAACACCRCGAAUUAAUCAUCCAUGCCAUGGAAGCAGCA<br>UAUGCCCGCCUGCAUCUAUCCAUGCAUGAUGGUGGAAGGUUUCGGACCAGGCUUCAUUCCCC
>MI0001488   MIR166
GUGGAAUGUUGUCUGGUUCAAGGUCUUGCUWUCCGAUUUGAGGAUGAUCCAUGCUUGCAUGU<br>GUAGUUUUUUUUGUUCCUCAGAUCUACAAGAUCUCGGACCAGGCUUCAUUCCCC
**G1:**
>MI0001493   MIR172
GCGUGGCAUCAUCAAGAUUCACAACCCAUCAAUCCGAACCACUGAUUUGGAAUGCAUGYAUGAGA<br>AUCUUGAUGAUGCUGCAU
>MI0005773   MIR396
UUCCACAGCUUUCUUGAACUUUCUUUUUCAUUUCCCUUAUUUUASAGCGAAAUUAAAUAACUAA<br>AAAUCUCUAACAUUUAACACUCUASAAAAAAAAAGCUCAASAAAGCUGUGGGA

***** *G2 contains no abnormal sequences.*

Table S3: Results of miRFam with uni-, bi- tri- and tetra-gram features on multiple-family datasets.

T_{20}	G_1	G_2	Total*
99.59%	100%	99.02%	98.24%

* Combination of T_{20}, G_1 and G_2.

All results are achieved by 5-fold cross validation.

Figure S1: Tree view of those four sequences misclassified by *miRFam* in the *let-7+R1* experiment.

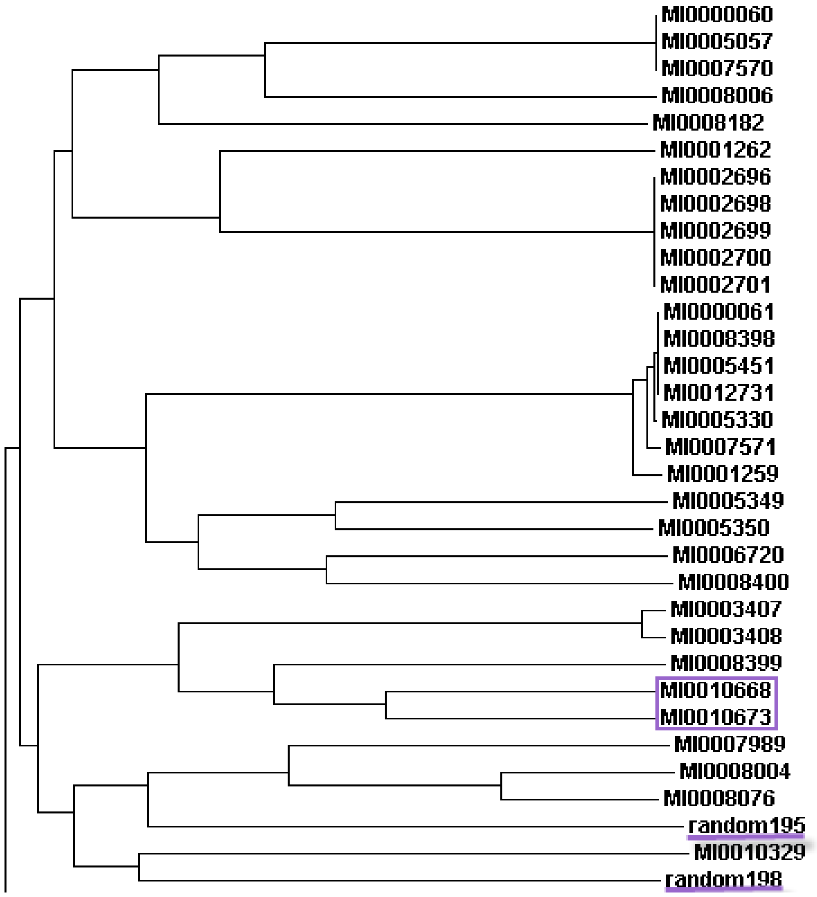

Since the whole tree contains 416 entries that is too big to show in a single page, here we only give the part related to misclassified entries. We got the multiple sequence alignment result by *Clustal W2* [2] and viewed the guide tree using *Jalview* [3].

We found that MI0010673 and MI0010668 are located in a separate branch, while RANDOM195 and RANDOM198 lie in the nearby branch. This means that these synthetic sequences are too similar to the real ones so that they are indistinguishable for SVM.

Figure S2: Cluster center comparison between three biggest miRNA families (let-7, mir-17 and mir-9) and synthetic datasets R1, R2 and R3. Here, only 80 (trigram & bigram) features are shown, since reversing a miRNA sequence can't change its base composition. The synthetic sequences are created by *squid* [4].

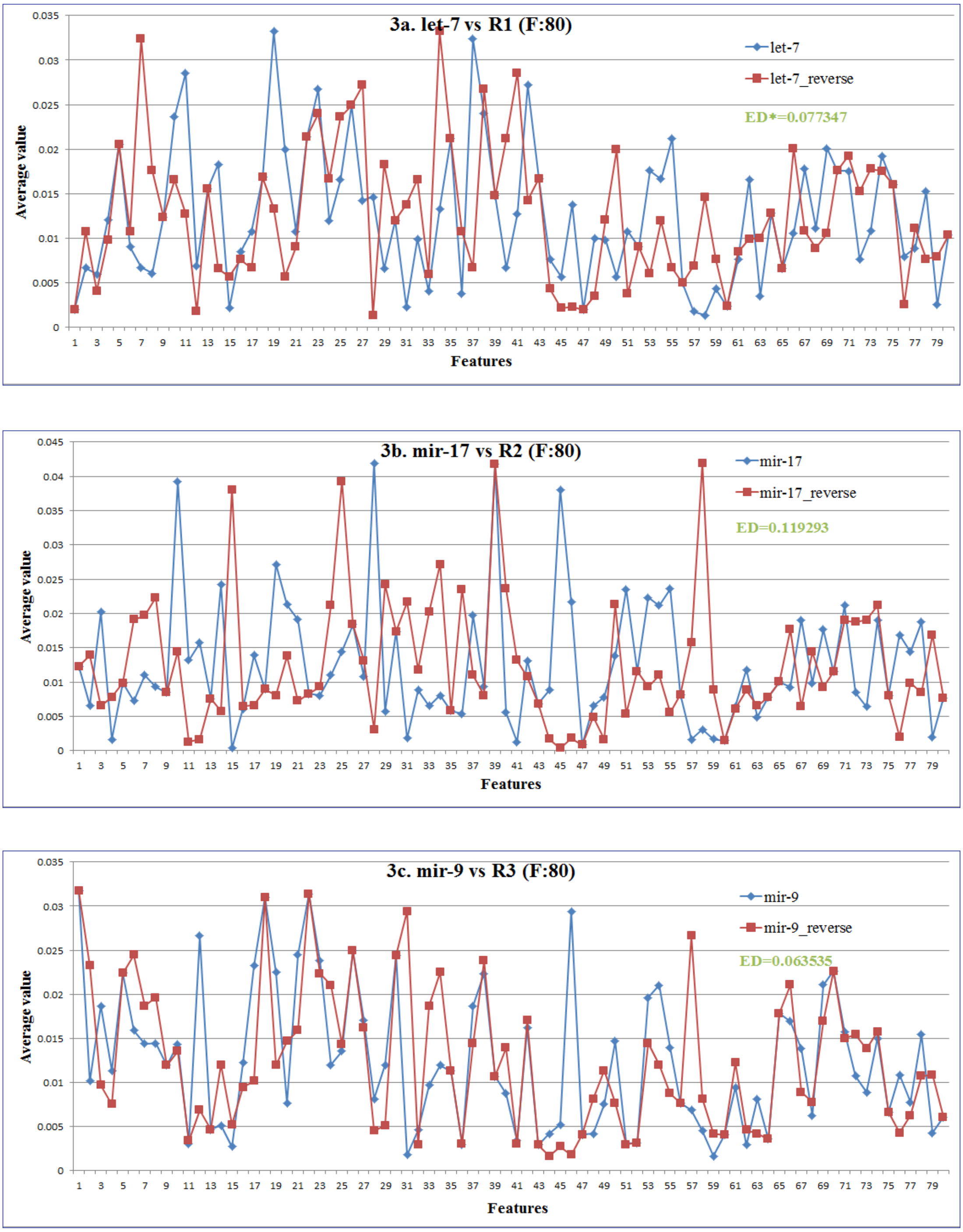

**ED* - Euclidean distance*

Figure S3: In miRBase, there are so many (around 60%) small families which have less than 5 members. But they only contain a little portion of all miRNAs registered in miRBase (around 15%). a.) family distribution in miRBase14.0. b.) family distribution in miRBase15.0. c.) sequence distribution in miRBase14.0. d.) sequence distribution in miRBase15.0.

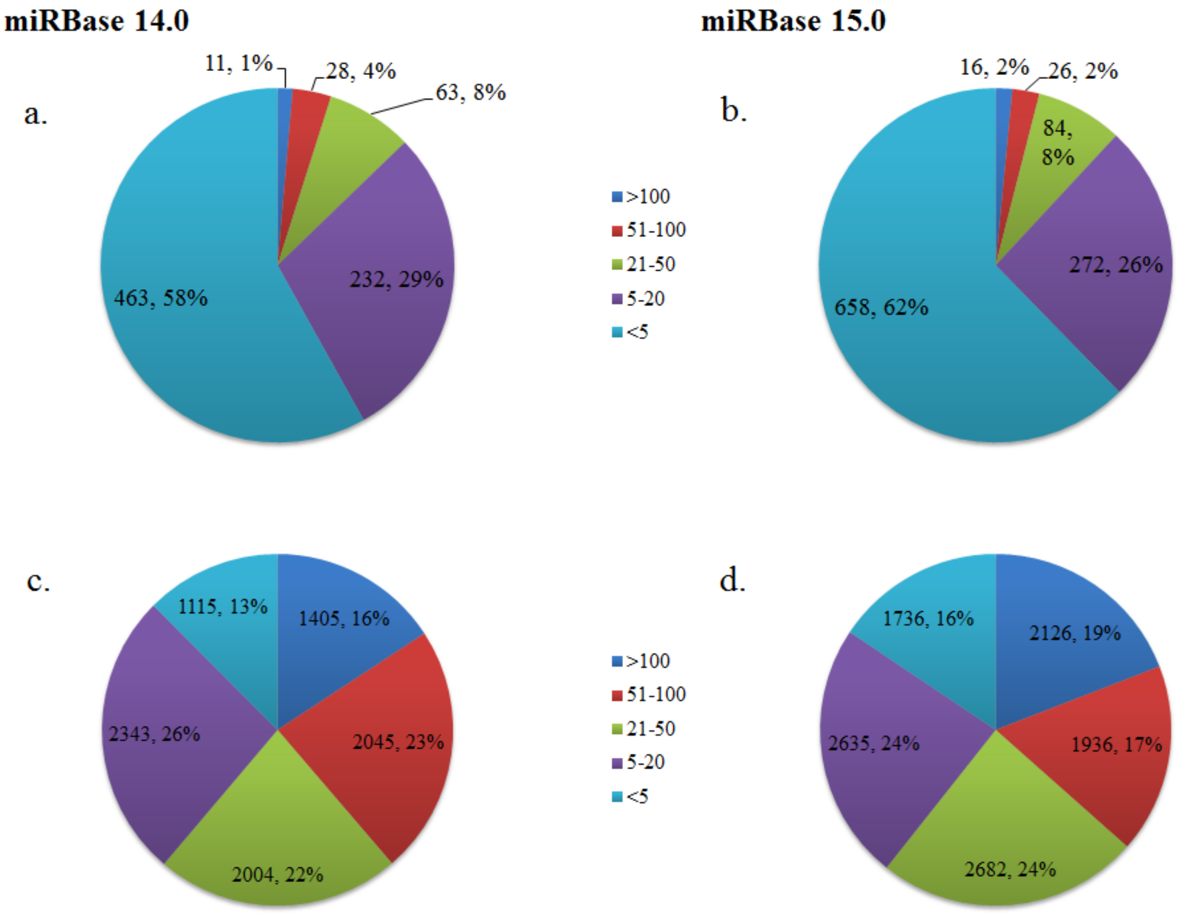

REFERENCES:

1. Griffiths-Jones S, Saini HK, van Dongen S, Enright AJ: **miRBase: tools for microRNA genomics.** *Nucleic acids research* 2008, **36**:D154-8.

2. Larkin MA, Blackshields G, Brown NP, et al.: **Clustal W and Clustal X version 2.0.** *Bioinformatics* 2007, **23**:2947-8.

3. Waterhouse AM, Procter JB, Martin DM, Clamp M, Barton GJ: **Jalview Version 2--a multiple sequence alignment editor and analysis workbench.** *Bioinformatics* 2009, **25**:1189-91.

4. Rivas E, Eddy SR: **Secondary structure alone is generally not statistically significant for the detection of noncoding RNAs.** *Bioinformatics* 2000, **16**:583-605.
